# The impact of Covid-19 on the Mental Health of the Portuguese Population

**DOI:** 10.1192/j.eurpsy.2022.1296

**Published:** 2022-09-01

**Authors:** I. Dos Santos, J. Saraiva, E. Mesquita, M. Coelho

**Affiliations:** 1 Psicobodycare, Psychology, Porto, Portugal; 2 Faculdade de Psicologia e de Ciências da Educação, Psychology, Porto, Portugal; 3 Casa de Acolhimento Sol Nascente, Statistic, Monte Córdova, Portugal

**Keywords:** Well-being, Covid-19, Mental Health

## Abstract

**Introduction:**

Covid-19 does not only have repercussions on the physical level, representing a new way of life, both individually and in society. The pandemic results in invisible consequences for the population’s mental health.

**Objectives:**

This study aimed to explore the consequences of Covid-19 on mental health in Portugal with a view to understanding and promoting the well-being and happiness of the Portuguese.

**Methods:**

The study included 111 young people and adults, aged between 18 and 58 years (M= 21.81, SD= 5.34), with 43.7% males and 52.7% females. A sociodemographic questionnaire was applied to all participants, as well as the Échelle de Mesure des Manifestations du Bien-Être Psychologique (ÈMMBEP; Massé et al., 1998 - Portuguese translation by Monteiro, Tavares & Pereira, 2012) which translates into a response scale 5-point Likert type, with five subscales, including happiness. In addition, a semistructured interview with data collection instruments was administered.

**Results:**

The results obtained demonstrate the negative impact of Covid-19 on the level of well-being, regardless of the participant’s gender or age.

**Conclusions:**

The data presented point to the need to sensitize individuals to the risk of the pandemic in terms of mental health, thus increasing society’s awareness of the psychological effects of this new global disease. Therefore, coping mechanisms are essential to promote well-being and successfully overcome the pandemic.

**Disclosure:**

No significant relationships.

